# Impact of a phage cocktail targeting *Escherichia coli* and *Enterococcus faecalis* as members of a gut bacterial consortium *in vitro* and *in vivo*

**DOI:** 10.3389/fmicb.2022.936083

**Published:** 2022-07-22

**Authors:** Colin Buttimer, Tom Sutton, Joan Colom, Ellen Murray, Pedro H. Bettio, Linda Smith, Andrei S. Bolocan, Andrey Shkoporov, Akihiko Oka, Bo Liu, Jeremy W. Herzog, R. Balfour Sartor, Lorraine A. Draper, R. Paul Ross, Colin Hill

**Affiliations:** ^1^APC Microbiome Ireland, University College Cork, Cork, Ireland; ^2^Center for Gastrointestinal Biology and Disease, Division of Gastroenterology and Hepatology, Department of Medicine, University of North Carolina at Chapel Hill, Chapel Hill, NC, United States; ^3^Department of Internal Medicine II, Faculty of Medicine, Shimane University, Izumo, Japan; ^4^School of Microbiology, University College Cork, Cork, Ireland

**Keywords:** bacteriophage, phage cocktail therapy, bacteria consortium, *Escherichia coli*, *Enterococcus faecalis*, gut, murine model, fermentation

## Abstract

Escherichia coli and *Enterococcus faecalis* have been implicated as important players in human gut health that have been associated with the onset of inflammatory bowel disease (IBD). Bacteriophage (phage) therapy has been used for decades to target pathogens as an alternative to antibiotics, but the ability of phage to shape complex bacterial consortia in the lower gastrointestinal tract is not clearly understood. We administered a cocktail of six phages (either viable or heat-inactivated) targeting pro-inflammatory *Escherichia coli* LF82 and *Enterococcus faecalis* OG1RF as members of a defined community in both a continuous fermenter and a murine colitis model. The two target strains were members of a six species simplified human microbiome consortium (SIHUMI-6). In a 72-h continuous fermentation, the phage cocktail caused a 1.1 and 1.5 log (log_10_ genome copies/mL) reduction in *E. faecalis* and *E. coli* numbers, respectively. This interaction was accompanied by changes in the numbers of other SIHUMI-6 members, with an increase of *Lactiplantibacillus plantarum* (1.7 log) and *Faecalibacterium prausnitzii* (1.8 log). However, in germ-free mice colonized by the same bacterial consortium, the same phage cocktail administered twice a week over nine weeks did not cause a significant reduction of the target strains. Mice treated with active or inactive phage had similar levels of pro-inflammatory cytokines (IFN-y/IL12p40) in unstimulated colorectal colonic strip cultures. However, histology scores of the murine lower GIT (cecum and distal colon) were lower in the viable phage-treated mice, suggesting that the phage cocktail did influence the functionality of the SIHUMI-6 consortium. For this study, we conclude that the observed potential of phages to reduce host populations in *in vitro* models did not translate to a similar outcome in an *in vivo* setting, with this effect likely brought about by the reduction of phage numbers during transit of the mouse GIT.

## Introduction

The human gastrointestinal tract (GIT) hosts a wide variety of microorganisms (bacteria, archaea, yeasts, protists, and viruses), with the number of bacteria residing in the human gut estimated to be 10^11^ CFU/g of feces (Shkoporov and Hill, 2019). Bacteria of the GIT play an essential role in producing and releasing vitamins, amino acids, and fatty acids from dietary compounds (Morowitz et al., 2011). They are involved in the breakdown of toxic compounds that reach the GIT from the diet (Den Besten et al., 2013). Both their direct interaction (e.g., cellular components) and indirect activities via their metabolites are implicated in the regulation of host immunity (Hill and Artis, 2010; Brestoff and Artis, 2013). Their presence in the GIT plays a vital role as a barrier against colonizing pathogenic bacteria (Belkaid and Hand, 2014). Furthermore, they are implicated in brain development, function and behavior modulation (Dinan et al., 2017). Disruptions of this bacterial community have been associated with poor nutrient utilization (Turnbaugh et al., 2006), impaired brain function (Fröhlich et al., 2016), immunity-related illnesses such as inflammatory bowel disease (IBD) (Duchmann et al., 1995), as well as diseases of the heart (Tang et al., 2017), liver (de Faria Ghetti et al., 2018), and kidneys (Chen et al., 2019).

Changes to the gut microbiome can occur as a result of different insults. Treatment with antibiotics causes a reduction in levels of bacterial diversity and changes in the relative abundance of individual species. Diversity often returns to baseline levels after-antibiotic treatment, but permanent changes can also result (Jernberg et al., 2010; Elvers et al., 2020). The use of antibiotics has been linked to an increased prevalence of antibiotic resistance genes within the gut microbiome and increased susceptibility to infections (Wilcox, 2003; Forslund et al., 2013). Moreover, their use is implicated with altered microbiome metabolism linked to obesity and the development of immunological disorders such as asthma and allergies (Sepp et al., 2005; Cho et al., 2012; Kuo et al., 2013). Dietary intake of protein, lipids, carbohydrates and polyphenols has also been shown to influence gut microbiome composition, seen as changes in the abundance of different taxa (Singh et al., 2017). Some probiotics are also designed to temporarily modulate gut microbiome functionality by supplementing the GIT with bacteria that produce metabolites or secrete substances (such as immunomodulation factors) that bring about health benefits (Preidis and Versalovic, 2009).

Many antibiotic or dietary approaches lack specificity and are not capable of targeted knockdown of selected bacterial members of the human gut community. One potential strategy to precisely target selected bacteria could be the use of bacteriophages (phages). Phages are viruses that specifically infect bacteria. Their numbers are estimated to be similar to that of their bacterial hosts in the human gut, and collectively, they are referred to as the gut virome. It is thought that the virome may shape bacterial communities (Hsu et al., 2019; Shkoporov and Hill, 2019). Their populations in the GIT are highly stable and highly individualized (Shkoporov et al., 2019). Changes in virome structure have correlated with non-alcoholic fatty liver disease (NAFLD) and IBD (Clooney et al., 2019; Lang et al., 2020). Nonetheless, their role, if any, in bacterial population structure or function remains unknown. The best evidence that phages may influence health outcomes is in fecal transplants, where treatment with filtered fecal filtrates was just as effective as non-filtered fecal transplants from healthy donors in treating *Clostridioides difficile* (Ott et al., 2017). Furthermore, studies using murine models have shown that filtered fecal filtrates can induce changes to the gut flora composition among receipt mice (Draper et al., 2020; Rasmussen et al., 2020).

Phages as therapeutics have been successfully employed for *Staphylococcus* and *Mycobacterium* infections, among many others (Fish et al., 2016; Dedrick et al., 2019). Still, their potential to target bacterial taxa in the gut has not been rigorously tested. Both *Escherichia coli* and *Enterococcus faecalis* have been implicated in the development of IBD, which includes both Crohn’s disease and ulcerative colitis. Thus, targeted knockdown of these species in the GIT without collateral damage to the microbiome could permit an improved understanding of their potential role in the pathogenesis of this condition(s) (Darfeuille-Michaud et al., 1998; Balish and Warner, 2002; Kotlowski et al., 2007; Miquel et al., 2010). Previous studies utilizing gnotobiotic mice have demonstrated the potential of phages to cause targeted knockdown of *E. coli* and *E. faecalis* in feces (Galtier et al., 2017; Duan et al., 2019). Phage predation of these species as part of a consortium could potentially influence the abundance of non-targeted community members (Hsu et al., 2019). However, most studies overlook the effect on non-target species or if induced changes are found to be transient in mammalian gut models (Draper et al., 2020; Javaudin et al., 2021).

Like with antibiotics, gut bacteria can become resistant to phage infection. This resistance can develop due to mutations affecting phage host cell receptors (Burmeister et al., 2020) or the insertion of spacers within CRISPR arrays which form part of the CRISPR/cas system targeting mobile genetic elements such a phage (Rasmussen et al., 2022). However, phages can also mutate to overcome such events causing bacteria and phages to be in a constant arms race of co-evolution (De Sordi et al., 2019).

In this study, we used both an *in vitro* and *in vivo* model to target *E. coli* and *E. faecalis* as part of a six-member bacterial consortium (SIHUMI-6) with a phage cocktail of four *Escherichia* and two *Enterococcus* phages. These included a continuous fermentation system and a gnotobiotic murine model designed to elicit mild colitis. Our results have significant implications for the translational value of these models and the future of using phages as a tool to engineer the microbiome. In brief, the ability of phage to partially reduce populations of target strains *in vitro* was not replicated *in vivo*. This effect was likely brought about by the reduction of phage numbers during transit of the mouse GIT, though a potential impact on the functionality of the consortium was observed.

## Materials and methods

### Bacteria cultivation, isolation, and identification

A list of bacterial isolates and phages utilized in this study is detailed in Supplementary Table S1. Unless otherwise stated, all isolates of *E. coli* and *E. faecalis* cultures were routinely grown on Lysogenic broth (LB, Merck, Darmstadt, Germany) and Tryptic soya broth (TSB, Merck), respectively. Either as liquid broth or agar plates (1% [w/v]) at 37°C. Isolation of novel bacterial isolates for phage host range analysis was obtained by diluting human feces in maximum recovery diluent and plating them onto either Bile Esuline azide agar (Merck) for isolation of *E. faecalis* and MacConkey agar (Merck) or Coliform agar (Merck) for isolation of *E. coli*. Identification of *E. faecalis* was achieved using a multiplex PCR using species-specific primers and *E. coli* by phylotyping PCR (Dutka-Malen et al., 1995; Clermont et al., 2013).

### Phage cultivation conditions, isolation, enumeration, and host range analysis

Phages were routinely cultivated at 37°C by the soft agar overlay method (Sambrook and Russell, 2001) using either LB or TSB as agar plates (1% [w/v]) and overlays (0.2% [w/v] agarose, Sigma-Aldrich, St. Louis, MO, United States) supplemented with 10 mM MgSO_4_. *E. faecalis* strain OG1RF and *E. coli* strain LF82 were used as the host to propagate *Enterococcus* phages A2 and Sw5 and *Escherichia* phages A4, I29, W115, and A51.2, respectively. All phages were originally isolated using an enrichment method using numerous sources. Either animal (A2, A4, A51.2)/human (I29) fecal matter or sewage (W115, Sw5) was placed into TSB or LB broth with an overnight culture of *E. faecalis* OG1RF or *E. coli* LF82, incubated overnight at 37°C with shaking, which was then centrifuged to pellet solid matter with supernatant then filtered (0.45 μm pore-size filter, Sarstedt, Nümbrecht, Germany). The supernatant was spotted onto overlays seeded with host strain. The phages were isolated by picking an individual plaque followed by replating and reisolating to ensure purity (Sambrook and Russell, 2001). Sodium chloride (NaCl)–magnesium sulfate (SM) buffer (50 mM Tris-HCl pH 7.5, 100 mM NaCl, 8 mM MgSO4) was used for serial dilutions for phage enumeration and host range analysis. Phage numeration and host range were performed by spot assay. This involved adding a hundred microliters of bacterial culture to 3 mL of the supplemented overlay (0.2% agarose), poured onto agar plates (1%) and let set. Ten microliters taken from ten-fold serial dilution of phage lysate were spotted onto the overlay and let dry before incubating overnight at 37°C followed by plaque enumeration the following day.

### Assessment of phage cocktail to prevent the formation of phage resistant

Either LB or TSB (supplement with 10 mM MgSO_4_) was inoculated with 1% with an overnight culture of host strain and allowed to grow to approximately 1 × 10^8^ CFU/mL. Phages were then added individually or as a cocktail to the culture to achieve a multiplicity of infection (MOI) of ether 0.2 or 0.5. This was then placed onto a microtiter plate reader (Multiskan FC, ThermoScientific, Waltham, MA, United States) with the following settings: incubation at 37°C continually, reading every hour for 48 h (total 49) at an absorbance of 594 nm with the pulse setting turned on for 10 s before reading.

### Transmission electron microscopy analysis

Phage lysate underwent centrifugation at 107,328 × g at 4°C for 2 h using an F65L-6 × 13.5 rotor (ThermoScientific, Waltham, MA, United States). The resulting phage pellets were resuspended in SM buffer. This phage preparation was then placed onto a CsCl step gradient composed of 1.3, 1.4, and 1.5 g/mL layers and spun at 107,328 × *g* for 4°C for 2.5 h using an F65L-6 × 13.5 rotor. The resulting bands were collected and desalted using Amicon Ultra-0.5 Centrifugal Filter Units with a 3 kDa MWCO membrane (Merck Millipore, Burlington, MA, United States). Following this, the purified phage preparation was placed onto a Formvar/Carbon 200 Mesh, Cu grids (Electron Microscopy Sciences, Hatfield, PA, United States) with subsequent removal of the excess sample by blotting. Grids were then negatively contrasted with 0.5% (w/v) uranyl acetate and examined at UCD Conway Imaging Core Facility (University College Dublin, Dublin, Ireland) by a transmission electron microscope.

### Phage DNA isolation and genome sequencing

A phage lysate was precipitated using polyethene glycol (15% w/v PEG8000, 1M NaCl) at 4°C overnight and centrifuged (11,000 × *g* at 4°C for 60 min), after which the pellet was resuspended in SM buffer. The resulting phage particles were then treated with DNase I (Thermo Fisher Scientific, Waltham, MA, United States) and RNase I (Thermo Fisher Scientific) before genome DNA extraction using a commercial phage DNA isolation kit (Norgen, Thorold, ON, Canada, Cat no. 45800) and subsequent clean up using a commercial DNA clean up kit (Promega, Cat no. A7280), as per the manufacturer’s protocol. DNA was then quantified using the Qubit broad range assay (Invitrogen/ThermoFisher Scientific) before standardizing paired end Nextera XT library preparation (Illumina, San Diego, CA, United States). Library quality was inspected with the Agilent Technology 2100 Bioanalyser using a High Sensitivity DNA chip before sequencing with an Illumina MiSeq platform (Illumina Inc., San Diego, CA, United States). Genome *de novo* assembly was performed using default parameters with MetaSPAdes (St. Petersburg, Russia).

### Phage genome annotation and phylogenetic analysis

The RAST server (Aziz et al., 2008) was employed with GLIMMER (Delcher et al., 1999) to predict open reading frames (ORFs). With further analysis of ORFs conducted with BLASTP, Pfam, (Finn et al., 2015), Interproscan, (Jones et al., 2014), and HHpred (Söding et al., 2005). With the detection of ORFs with transmembrane domains, signal peptide sequence and lipoproteins being identified with the use of TMHMM v.2 (Krogh et al., 2001), SignalPv.4.1, (Petersen et al., 2011), and LipoP v.1 (Juncker et al., 2003), respectively. Translated ORFs from phages were searched against hidden Markov model profiles downloaded from the Prokaryotic Virus Orthologous Groups (pVOGs) database (Grazziotin et al., 2017) using hmmscan (v.3.1b2) (Eddy, 2011) with an *E*-value cut off of 1 × 10-5. The presence of transfer RNA genes was investigated using ARAGORN (Laslett and Canback, 2004).

Genome comparison between phages was visualized with the use of Easyfig (Sullivan et al., 2011), with comparisons between genomes being made with TBLASTX. A total proteome comparison between phages was conducted using Coregenes 3.5 with the BLASTP threshold set at 75% (Turner et al., 2013). A heat map comparing the genomes was generated using Gegenees (Ågren et al., 2012), using TBLASTX or BLASTN with accurate parameters (fragment length: 200 bp; step size: 100 bp; threshold: 0%). VICTOR was employed for all pairwise comparisons between phages at the amino acid level, which uses the Genome-BLAST Distance Phylogeny (GBDP) method (Meier-Kolthoff et al., 2013) under settings recommended for prokaryotic viruses (Meier-Kolthoff and Göker, 2017). The resulting intergenomic distances (including 100 replicates each) were used to infer a balanced minimum evolution tree with branch support via FASTME, including SPR postprocessing (Lefort et al., 2015) for each formula D0, D4, and D6, respectively. The phylogenetic trees (with branch support) were rooted at the midpoint and visualized with iTOL (Letunic and Bork, 2019).

### Phage infection in a continuous fermentation with SIHIUMI-6

The SIHUMI-6 community was composed of *E. coli* LF82, *E. faecalis* OG1RF, *Bacteroides vulgatus* DSM1447, *Feacalibacterium prausnitzii* A2-165, *Bifidobacterium longum* ATCC15707 and *Lactiplantibacillus plantarum* WCFS1. A similar bacterial consortium was utilized previously by Eun et al. (2014), minus *Ruminococcus gnavus*. We were concerned that *R. gnavus* would contribute to colitis since it induces a high IFNg signal *ex vivo*, potentially masking any effect brought about by the reduction in *E. coli* LF82 levels. YCFA-GSCM broth was prepared as described elsewhere (Guerin et al., 2018) and used for the growth of the SIHUMI-6 community. The host range of EE phages on SIHUMI-6 was checked by spot assay using YCFA-GSCM agar (1% [w/v]) and overlay (agarose, 1% [w/v]). All fermentations were performed in a continuous format for four days with controlled conditions in a final volume of 200 mL and using myControl Minibio-500mL systems (Applikon biotechnology, Delft, Zuid-Holland, The Netherlands). The dissolved oxygen level was maintained below 0.1% by sparging with anaerobic gas mix (80% (v/v) N_2_, 10% (v/v) CO_2_, 10% (v/v) H_2_). The media was kept at 37°C with constant stirring at 50 rpm at a pH of 6.8. Vessels were inoculated with SIHUMI-6 strains (1% [v/v]), incubated for 24 h in a batch format, with the system subsequently swapped to continuous format with fresh broth pumped to the vessels at a rate of 400 mL/day. The treatment vessel was supplemented with 20 mL of a phage cocktail (approx. titer 1 × 10^8^ PFU/mL), while the control vessel was supplemented with a heat-killed phage cocktail. Samples were taken at 0, 24, 48, 72, and 96 h.

### qPCR for bacteria and phage quantification

Each bacterial strain and phage were quantified using bacteria and phage specific primers (Supplementary Table S2) using the SensiFAST SYBR No-ROX Kit (Bioline) and Roche LightCycler 480. For each well on a 96-well optical qPCR plate, a reaction mixture consisted of 7.5 μL of SYBR, 0.3 μL of the forward primer (μM), 0.3 μL reverse primer (μM) and 3.9 μL of water with 1 μL of template DNA (5 ng/μL). The thermocycling protocol consisted of initial denaturation at 95°C for 5 min, then 45 cycles of 95°C for 20 s, 60°C (or 62°C) for 20 s and 72°C for 20 s. Standard curves were included in every assay plate, consisting of at least five points. Each standard and sample were measured with three technical replicates. Standards consisted of a construct of the TOPO vector and the region being targeted on the phage/bacteria genome by qPCR. Standards were created using the TOPO TA Cloning Kit (Invitrogen, Waltham, MA, United States) using Mach1™-T1R One-Shot Cells (Invitrogen, Waltham, MA, United States) following the manufacturer’s instructions. GraphPad Prism (v8) was used to determine the normality of data using the D’Agostino-Pearson normality test between treatment groups at different time points. A comparison of parametrically distributed data was performed using a two-tailed unpaired *t*-test. Differences between bacterial consortia of fermentation vessels treatment groups were calculated using Bray Curtis dissimilarity (Bray and Curtis, 1957).

### Assessing the level of phage resistance among phage exposed host strain

Samples (fermentation/stool) were plated onto LB agar (1% [w/v]) supplemented with 100 μg/mL ampicillin for the selection of *E. coli* LF82 or TSB agar (1% [w/v]) with 50 μg/mL rifampicin for the selection of *E. faecalis* OG1RF. Colonies were selected and streaked out onto fresh LB/TSB agar plates without antibiotics. Overnight cultures were made using LB/TSB both and incubated at 37°C. These were then streaked across LB/TSB agar and allowed to dry. Streaks of phage lysates (c. 1 × 10^8^ PFU/mL) were then streaked perpendicular to these and allowed to dry before incubation overnight at 37°C. The intersection of phage and bacterial culture streaks were examined for the presence or absence of a contiguous line of bacterial growth indicating susceptibility or resistance, respectively. Cultures that were identified as resistant were then further tested by spot assay.

### Murine model setup

8-12 week-age germ-free (GF) 129SvEv background interleukin 10 deficient (*Il10*^–/–^) mice were obtained from UNC National Gnotobiotic Rodent Resource Center and housed within gnotobiotic isolators (Kim et al., 2005; Liu et al., 2011; Eun et al., 2014; Mishima et al., 2015; Oka et al., 2019). GF mice were colonized with SIHUMI-6 strains by oral gavages at day 0 and day 3 (Darfeuille-Michaud et al., 2004; Kim et al., 2005; Eun et al., 2014). The therapeutic gavage with heat-killed or live EE phages (>1 × 10^10^ PFU/mL phages in 200 μL) was performed from day seven twice a week for 9 weeks.

### Assessment of pro-inflammatory cytokines, stool lipocalin-2 and histology study of mice

Tissue fragment culture: Colonic tissue fragment cultures were prepared from the large intestine and the cecum as previously described (Liu et al., 2011; Mishima et al., 2015). In brief, colonic tissue was thoroughly irrigated with PBS, shaken at room temperature in RPMI containing 50 μg/mL gentamicin for 30 min at 250 rpm, cut into 1 cm fragments, dried up with paper, and weighed. Colonic tissue fragments were distributed (around 50 mg/well) into 24 well plates (Costar) and incubated in 1 mL of RPMI supplemented with 5% fetal bovine serum, 50 μg/mL gentamicin and 1% antibiotic/antimycotic (penicillin/streptomycin/amphotericin B, Gibco, Waltham, MA, United States) for 20 h at 37°C in a CO_2_ incubator. Following tissue culture, supernatants were collected for cytokine quantification by ELISA according to the manufacturer’s instructions (IFN-γ and IL-12p40 non-allele specific, R&D Systems, Minneapolis, MN, United States). The ELISA data were normalized to 50 mg of tissue weight.

Fecal lipocalin-2: Quantification of fecal lipocalin-2 was previously described (Oka et al., 2019). In brief, fresh feces were collected and stored at -80°C. Fecal samples (10-20 mg) were incubated overnight at 4°C in PBS containing 0.1% Tween 20 (Fisher Scientific) and vortexed shortly to obtain homogenous fecal suspensions. The fecal suspensions were centrifuged for 10 min at 12,000 rpm at 4°C. Clear supernatants were collected and stored at -20°C. ELISA were performed according to the manufacturer’s instruction (Mouse Lipocalin-2 DuoSet, R&D Systems, Minneapolis, MN, United States).

Histology analysis: The intestines were fixed in 10% neutral-buffered formalin. Paraffin-embedded sections (5 μm) were stained with hematoxylin and eosin by the Histology Core of the Center for Gastrointestinal Biology and Disease at UNC. The inflammation of the duodenum, jejunum, ileum, cecum, colon, and rectum was quantitated by two researchers at UNC in a blinded fashion as follows: score 0; no inflammation, and/or no epithelial hyperplasia, and/or goblet cells: present, score 1; infiltrating cells: some in lamina propria/focal to partially generalized, and/or epithelia hyperplasia: minimal, and/or goblet cells: present, score 2; infiltrating cells: more cells and more involvement, and/or epithelia hyperplasia: obvious but not marked, and/or goblet cells: mild loss, score 3; infiltrating cells: pronounced, entire sections involved, some involvement of submucosa and muscularis, and/or epithelia hyperplasia: marked, and/or goblet cells: moderate to marked loss, score 4; crypt abscess, and/or ulcers, and/or transmural inflammation – may extend into serosa (Rath et al., 1996; Liu et al., 2011).

### Enumeration of host strains and phage in mouse stool samples

Fecal samples were collected and weighted. For the enumeration of host strains, stool samples where serially diluted in PBS and plated either on LB agar (1% w/v) with ampicillin 100 μg/mL or TSB agar (1% w/v) with 50 μg/mL rifampicin and incubated at 37°C. GraphPad Prism (v8) was used to determine the normality of data using the Shapiro–Wilk test. A two-tailed unpaired *t*-test was used to compare parametrically distributed data. For comparisons using data found to be non-parametrically distributed, the Mann–Whitney test was utilized. For the enumeration of phage, stool samples were first diluted in SM buffer and filter sterilized (0.45 μm) with the subsequent creation of 10-fold serial dilution. Hundred microliters of a host culture was added to 3 mL of the supplemented overlay (0.2% agarose), poured onto agar plates (1%) and let set. Ten microliters taken from the dilution series were then spotted onto the overlay and let dry before incubating overnight at 37^°^C, followed by plaque enumeration the next day. The limit of detection for phage quantification was 5.4 × 10^3^ PFU/g.

### DNA extraction and preparation for metagenomic sequencing

The QIAamp Fast DNA Stool Mini Kit (Qiagen, Hilden, Germany) was used following the manufacturer’s protocol for the extraction of total DNA from fecal and luminol samples. Before extraction, the ZymobIOMICs spike-in control 1 (Zymo Research, Irvine, CA, United States) was added, 20 μL for every 20 mg of feces. Extracted DNA was measured using the Qubit High Sensitivity assay (Thermo Fisher Scientific) before standardization for paired-end Nextera XT library preparation (Illumina, San Diego, CA, United States). Library quality was inspected with the 4200 Agilent Tapestation (Agilent Technologies, Santa Clara, CA, United States) using the High Sensitivity D1000 ScreenTape assay (Agilent Technologies) before sequencing with an Illumina MiSeq platform (Illumina Inc., San Diego, CA, United States).

### Pre-processing of metagenomics reads

Raw sequence reads (2,889,583 ± 1,822,775 [mean ± SD]) derived from mouse feces were quality assessed using FastQC (v0.11.8). Nextera adapters were removed using Cutadapt (Martin, 2011), and reads were subsequently trimmed and filtered using Trimmomatic (v0.36) (Bolger et al., 2014) with the following parameters; HEADCROP:20 CROP:115 SLIDINGWINDOW:4:20 MINLEN:20. Mammalian reads were removed using Kraken (v1.0) (Wood and Salzberg, 2014) and an in-house database of the following genomes; GRCh38 human genome, *Mus musculus* strain C57BL/6J (NC_000067.6), *Sus scrofa* isolate TJ Tabasco breed Duroc (NC_010443.5) *Macaca mulatta* isolate 17573 (NC_027893.1), resulting in 2277480 ± 1500907 reads (mean ± SD) per sample.

### Read alignment and absolute abundance calculation

Trimmed and quality-filtered reads were aligned to a database of phage cocktail, SIHUMI-6 and spike-in genomes (*Imtechella halotolerans*, *Allobacillus halotolerans*) of the ZymoBIOMICS™ Spike-in Control I (High Microbial Load) using Bowtie2 (Langmead and Salzberg, 2012). Read alignments were processed using SAMtools (Li et al., 2009) to generate a count matrix for each sample. In order to filter out spurious read alignments to shared features across genomes and subsequent false positive detection of these genomes in samples, genomes that recruited less than 20 reads per sample were excluded. This figure was established by comparing the number of reads aligned to the genome *Lactococcus* phage Q33 in each sample (Mahony et al., 2013). Q33 was added as a control as it lacks a host in the community and would have passed through the mouse without replicating. At most, Q33 recruited 15 reads in one sample; hence the threshold was set to a minimum of 20 reads. In order to calculate the absolute abundance of phage and bacterial genomes in each sample, read counts were normalized per kbp of genome per million mapped reads (RPKM). The total cell/phage count per sample was calculated using each spike-in genome individually, multiplied by the number of cells added (2 × 10^7^) per the manufacturer’s instructions. Absolute counts for each phage and bacterial genome per sample were calculated using each genome and the resulting average total cell/phage. Statistics and figures for the mouse model were carried out in the R environment (v4.1.0). Tests for significance between phage and host abundance groups were performed using the Wilcoxon test, with significance defined as less than *p* < 0.05.

### Access to genomic sequence data of study

Genomes of the phages described in this study are available at GenBank under accession numbers given in Table 1. Sequencing data reported in this paper is deposited in the Sequence Read Archive (SRA) of the National Centre for Biotechnology Information (NCBI) under BioProject ID PRJNA828263.

**TABLE 1:** GenBank submission numbers for phages of study.

Phage		Accession number
*Enterococcus* phage	A2	MT856905
	Sw5	ON286976
*Escherichia* phage	A4	ON286972
	I29	ON286975
	W115	ON286974
	A51.2	ON286973

## Results

### Isolation and characterization of phages against *Enterococcus faecalis* OG1RF and *Escherichia coli* LF82

**FIGURE 1:**
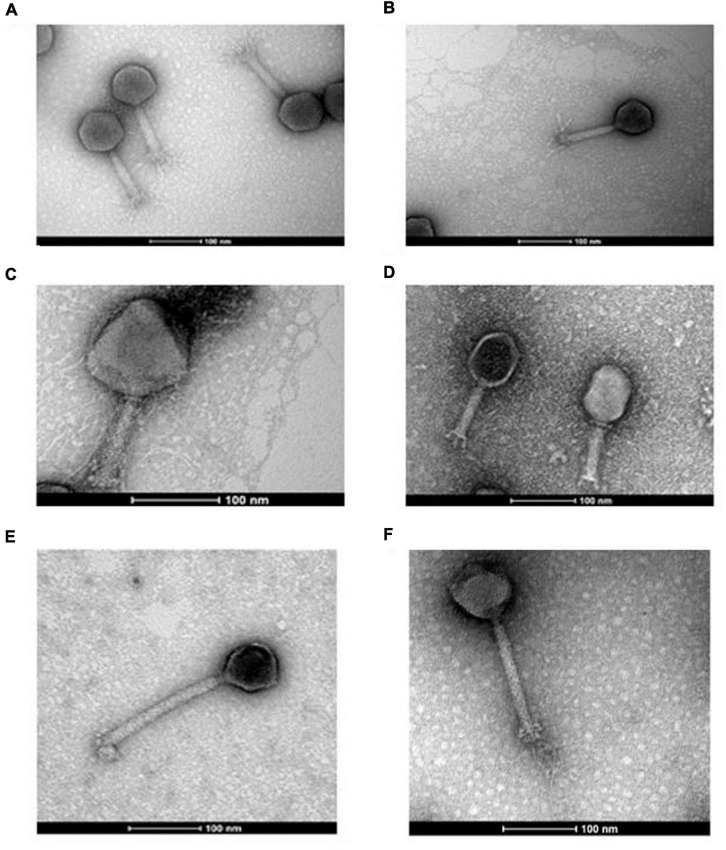
Transmission electron micrographs of negatively stained *Escherichia* phages I29 **(A)**, A51.2 **(B)**, A4 **(C)**, and W115 **(D)** and *Enterococcus* phages Sw5 **(E)** and A2 **(F)**. Uranyl acetate (0.5%) was used for negative staining. The scale represents 100 nm.

The phage utilized in this study were isolated from feces (animal or human) or sewage water (Supplementary Table S3). The bacterial hosts used for isolation and routine propagation were *E. faecalis* OG1RF (a rifampicin and fusidic acid-resistant derivative of strain OG1) isolated initially from oral samples, and *E. coli* LF82, the prototype strain of the adherent/invasive *E. coli* strain pathovar (AIEC), originally isolated from an individual suffering from Crohn’s disease (Gold et al., 1975; Dunny et al., 1978; Miquel et al., 2010). All phages formed clear plaques on their respective hosts with diameters typically ranging between 0.5 and 2 mm on overlays using 0.2% w/v agarose (Supplementary Figure S1). Phages were visualized using transmission electron microscopy, and all were found to possess a myovirus-like morphology with capsids and contractile tails with four distinct virion structures (Figure 1). Dimensions of phage virions are detailed in Supplementary Table S4.

All six phages were sequenced, revealing genomes sizes ranging from 135 – 355 kbp (Table 2). No genes were identified that could encode for integrases, excisionases or repressor proteins, suggesting that all six phages possess an exclusively lytic lifecycle. Look to Supplementary Tables S5–S10 for genome annotation of these phages. The virulent nature of these phages was also supported by phylogenetic analysis (Supplementary Figures S2, S3), placing these phages among genera of *Vequintavirus* (phages I29 and A51.2), *Krischvirus* (phage W115), *Schiekvirus* (phage Sw5) and *Kochikohdavirus* (phage A2), with *Escherichia* phage A4 identified as a relative of *Escherichia* phage PBEC04 (member of the genus *Asteriusvirus*). Previously described phages belonging to these taxonomic classifications follow a strictly lytic lifecycle (Denou et al., 2009; Dalmasso et al., 2016; Buttimer et al., 2017; D’Andrea et al., 2019).

**TABLE 2:** Summary of genomic features of phages used in this study.

Phage	Taxonomic assignment	Relative (accession no.)	Genome size (bp)	*ORF*s	tRNA genes	coverage
*Escherichia* phage I29	*Myoviridae/Vequintavirinae/Vequintavirus*	*Escherichia* phage rV5 (DQ832317.1)	137,044	214	7	487
*Escherichia* phage A51.2	*Myoviridae/Vequintavirinae/Vequintavirus*	*Escherichia* phage rV5 (DQ832317.1)	137,300	215	7	447
*Escherichia* phage A4	*Myoviridae*/Rak2-like	*Escherichia* phage PBECO4 (KC295538.1)	355,528	591	6	98
*Escherichia* phage W115	*Myoviridae/Tevenvirinae/Krischvirus*	*Escherichia* phage RB49 (AY343333.1)	163,997	268	0	109
*Enterococcus* phage A2	Herelleviridae/Brockvirinae/*Schiekvirus*	*Enterococcus* phage EFLK1 (KR049063)	149,431	191	24	344
*Enterococcus* phage Sw5	*Herelleviridae/Brockvirinae/Kochikohdavirus*	*Enterococcus* phage phiEF24C (AP009390.1)	143,759	212	8	260

One of the goals of this study was to assess the potential for using phages infecting species of *E. coli* and *E. faecalis* to elicit a targeted knocked of these species due to their potential role in the pathophysiology of IBD. A host range was conducted that included isolates of these species obtained from the feces of individuals with IBD. The identity of these strains was verified by *E. coli* phylotyping and multiplex PCR for *Enterococcus* speciation (Supplementary Table S1).

**FIGURE 2:**
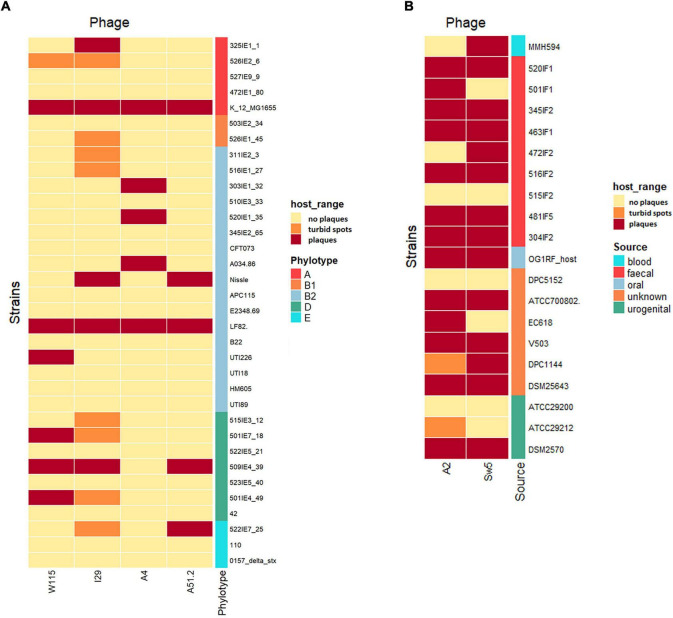
Host range of **(A)** *Escherichia* phage and **(B)** *Enterococcus* (*x*-axis) phage on different strains of *E. coli* or *E. faecalis* (*y*-axis) presented as heatmaps, respectively.

*Escherichia coli* is a genetically diverse species with seven distinct phylogenetic clades (A, B1, B2, C, D, E, and F). Our host range analysis of *Escherichia* phages utilized 34 isolates encompassing phylogroups A, B1, B2, D, and E. The four phages were able to form plaques on 12 of the 34 isolates (35%), including six of the 17 (35%) isolates obtained from individuals with IBD (Figure 2A). Among the IBD strains, the efficiency of plating (EOP) values ranged from 1 × 10^–4^ to 1.9 (Supplementary Table S11). Over the entire collection of *E. coli* strains, phages plaqued on six of the 17 B2 (35%) and three of the seven (43%) D phylogroup isolates. They were also capable of plaquing on *E. coli* belonging to phylogroups A and E. Additionally, phage I29 and W115 could generate zones of clearing on several other strains but did not form clear plaques. The host range of the *Enterococcus* phages was assessed on 20 isolates of *E. faecalis*, including isolates obtained from the feces of individuals with IBD. These phages were found to have a relatively broad host range, with phages Sw5 and A2 forming plaques on 13 and 14 of the total isolates, respectively (Figure 2B). Both phages could form plaques on *E. faecalis* obtained from individuals with IBD, both infecting seven of these nine isolates where EOP values ranged between 0.006 to 82 (Supplementary Table S12). As observed with the *Escherichia* phages, phage Sw5 could also inhibit the growth of two isolates in spot assays but without plaque production.

**FIGURE 3:**
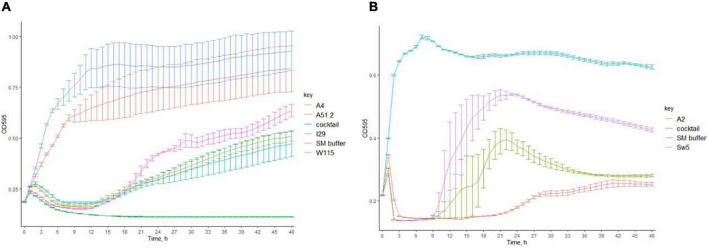
The ability of **(A)** *Escherichia* phages (MOI of 0.5) and **(B)** *Enterococcus* phages (MOI of 0.2) individually or in cocktails to inhibit the formation of phage resistant of *E. coli* LF82 and *E. faecalis* OG1RF (bars represent mean ± SD).

We investigated the ability of the *Escherichia* and *Enterococcus* phage to prevent the emergence of phage resistance either on their own or as part of a cocktail. Individually, three of four of the *Escherichia* phages (MOI of 0.5) could cause lysis on *E. coli* LF82 (Figures 3A,B). However, re-growth occurred after infection with these *Escherichia* phages approximately 21 h after lysis, suggesting the emergence of phage resistance among the host strain. The fourth phage, *Escherichia* phage A4, could not cause lysis of its host in broth. A cocktail composed of all four *Escherichia* phages could prevent the emergence of phage resistance. Furthermore, the two *Enterococcus* phages (MOI of 0.2) could not prevent re-growth following initial host lysis, either on their own or as part of a cocktail.

A high titer cocktail (>1 × 10^10^ PFU/mL in SM buffer) of these six phages was inspected for their long-term stability by spot assay. Storage over two years at 4°C resulted in a relatively small decrease in titer of between 1.34 log and 0.04 log for *E. coli* and *E. faecalis* phages, respectively (Supplementary Figure S4).

### Impact of phages in a continuous fermentation of the SIHUMI-6 consortium

We initiated a continuous culture with a SIHUMI-6 consortium consisting of the *E. coli* and *E. faecalis* target strains, together with common gut commensals including *Lactiplantibacillus plantarum*, *Bacteroides vulgatus*, *Faecalibacterium prausnitzii*, and *Bifidobacterium longum*. Prior to this fermentation, the ability of these six phages to infect other members of the SIHUMI-6 consortium was assessed. Look to Supplementary Figure S5 for an illustration describing the experiment setup. Using a spot assay, a 5 μL volume of each phage lysate (≥1 × 10^7^ PFU/mL) was tested on each species. After incubation in anaerobic conditions at 37°C, the phage cocktail only formed spots on the target hosts.

**FIGURE 4:**
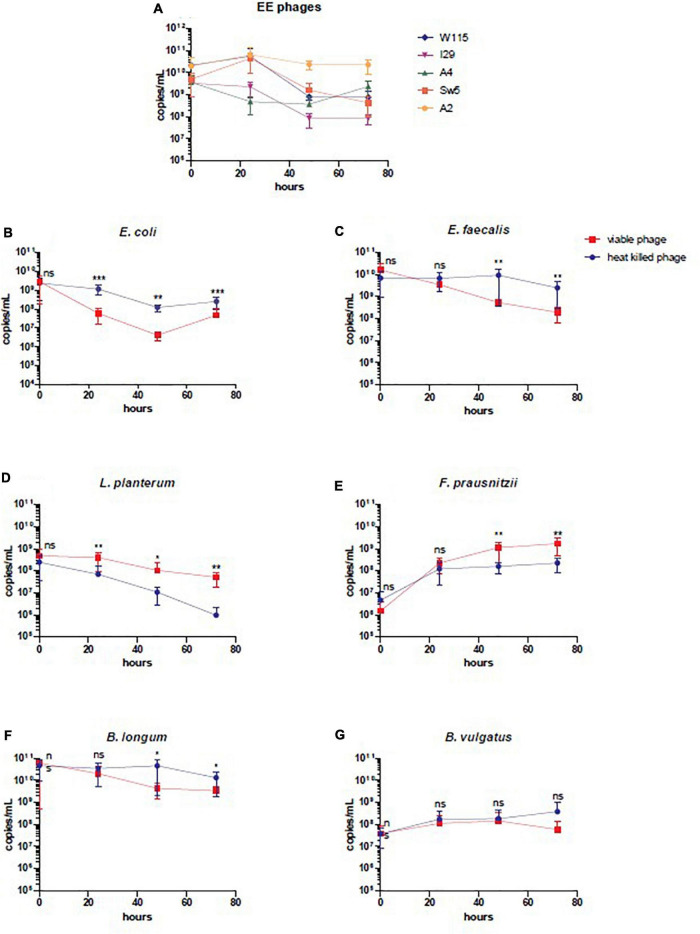
Enumeration by qPCR of individual members of SIHUMI-6 consortium and EE phages. **(A)** EE phages, **(B)** *E. coli* LF82, **(C)** *E. faecalis* OG1RF, **(D)** *L. plantarum*, **(E)** *F. prausnitzii*, **(F)** *B. longum*, and **(G)** *B. vulgatus*, during continuous fermentation at different time points treated with heat-killed or viable phage (bars represent mean ± SD). Statistical significance was recorded as follows, not significant (ns), *p < 0.5, **p < 0.01**, and ***p < 0.001.

**FIGURE 5:**
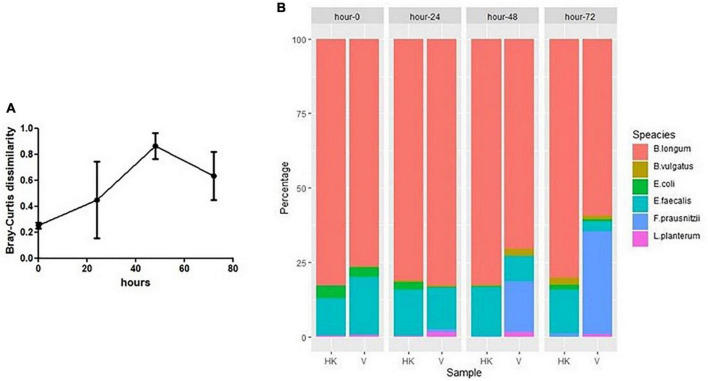
**(A)** Dissimilarity (Bray–Curtis) between SIHUIM-6 consortium treated with heat-killed or viable phage (bars represent mean ± SD). **(B)** Composition of SIHUMI-6 consortium among samples taken from continuous fermentations treated with heat-killed (HK) or viable (V) phages at 0, 24, 48, and 72 h, with mean quantities (genomes copies/mL) of each species illustrated as a percentage of the total population of the community.

In a continuous culture (chemostat culture), the growth rate of a bacteria/phage μ (h^–1^) is related to the dilution rate *D* (= *F/V* [h^–1^]), where *F* is the feed rate and *V* is the culture volume. Therefore, a bacterium or phage with μ < *D* will be diluted out of the fermentation vessel (Tanji et al., 2005). Quantification of phage by qPCR in the viable phage treated vessel confirmed that all six phages remained present throughout the experiment, ensuring that all could successfully propagate in this setting (Figure 4A). This finding agrees with spot assay counts on *E. coli* LF82 and *E. faecalis* OR1RF for enumeration of phages infecting these species (Supplementary Figure S6). Additionally, qPCR shows that SIHUMI-6 members remained present throughout the continuous fermentation in both treated and untreated vessels, albeit at different levels (Figures 4B–G). However, the presence of viable phages caused changes in the levels of five of the six members of the consortium. Before the addition of the EE phage cocktail, no significant difference was identified among the SIHUMI-6 consortia in both test and control fermenters. However, as the fermentation progressed, there was a reduction in the levels of *E. coli* LF82 in the test vessel after 72 h, with a 1.5 log fold difference (*p* < 0.01) in genome copies/mL and a 1.1 log fold difference (*p* < 0.05) in the levels of *E. faecalis* OG1RF at 72 h. The levels of three non-targeted members of the SIHUMI-6 were also affected. After 72 h, *L. plantarum* WCFS1 and *F. prausnitzii* A2-165 were 1.7 and 1.8 (*p* < 0.01) log fold higher in the test vessel, respectively. Finally, there was almost a 1 log decrease in *B. longum* (*p* < 0.05) in the test vessel. Obviously, these results confirm that the phage cocktail has a significant and reproducible effect on community composition. Comparison of the dissimilarity (Bray–Curtis) of the SIHUMI-6 consortia showed changes as each fermentation progressed for those treated with viable or heat-killed EE phage cocktails. This effect is illustrated by the increased dissimilarity of the SIHUMI-6 consortia among vessels treated with viable and heat-killed phages from the start point of fermentation (Figure 5A), showing a clear difference in the composition of these communities (most pronounced with *E. faecalis* and *F. prausnitzii*) at 48 and 72 h post-continuous fermentation (Figure 5B). Though, changes in the composition of the bacterial consortium could be detected for each treatment group as continuous fermentation progressed. The most prominent dissimilarity developed among the viable phage treated consortia over the duration of the experiment (Supplementary Figure S7).

**FIGURE 6:**
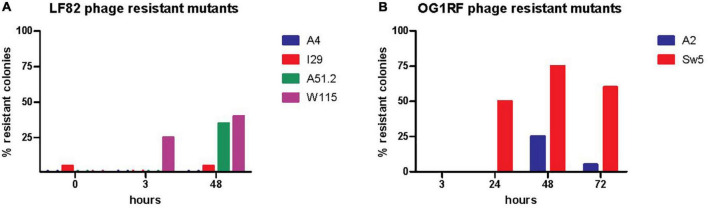
Assessing the presence of phage resistant of **(A)** *E. coli* LF82 and **(B)** *E. faecalis* OG1RF as part of the SIHUMI-6 consortium when targeted by EE phage cocktail throughout a 72 h continuous fermentation.

We also monitored the development of phage resistance in both *E. coli* LF82 and *E. faecalis* OG1RF over the 72 h fermentation. The highest density of phage host species was found at the initial stages of continuous fermentation, with reduced levels occurring at later stages of the experiment (Figures 4B,C). The level of host resistance differed between phage types, but there appeared to be an increased abundance of resistance against particular phages after the initial inspected time point of continuous fermentation (Figure 6). At hours 0, 3, and 48, the proportions of tested colonies resistant to *Escherichia* phage W115 were 0, 25, and 40%, respectively. No resistance was observed against *Escherichia* phage A51.2 at earlier time points, but 35% of tested colonies at hour 48 were resistant. This phage resistance emergence may explain the increased increase (1.05 log) of *E. coli* levels at 72 h. No or limited phage resistance was seen against *Escherichia* phage A4 and I29. Resistance was also observed among *Enterococcus* phage A2 with 0, 0, 25, 5% and Sw5 with 0, 50, 75, and 60% of tested colonies resistant at hours 3, 24, 48 and 72, respectively.

### Impact of phage in a murine model colonized with the SIHUMI-6 consortium

A murine trial was conducted to assess the performance of the EE phage cocktail against targeted *E. coli* and *E. faecalis* as part of a six-member bacterial consortium within the GIT. Look to Supplementary Figure S8 for an illustration describing the experiment setup. Inspection of stool samples by selective plating for *E. coli* LF82 and *E. faecalis* OG1RF indicated no significant changes in levels between test and control cohorts at the time points inspected and over the duration of the test (Supplementary Figure S9).

**FIGURE 7:**
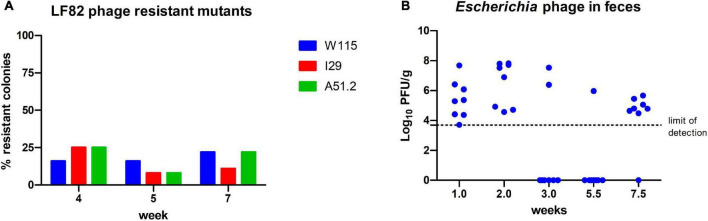
Enumeration **(A)** *E. coli* LF82 phage resistant colonies and **(B)** viable EE phages among feces at different time points of the viable phage treated mice.

We examined *E. coli* LF82 isolated from the feces of mice (*n* = 3) treated with viable phage at selected time points (week: 2, 5, and 7) throughout the trial, where 11–33% of isolated colonies were found insensitive to infection by phages W115, 129 and A51.2. No resistance was observed among colonies of *E. coli* LF82 obtained from the feces of the cohort administered heat treated phages at equivalent time points (Figure 7A). Phage resistance was not found among inspected mice (*n* = 3) of viable phage treated cohort for *E. faecalis* OG1RF colonies inspected over-tested time points (week: 1 and 8.5). Additionally, it was possible to detect *Escherichia* phages (5.4 × 10^3^ – 6.6 × 10^7^ PFU/g [range]) in 46% (26/56) of inspected stool samples of the viable phage treated cohort (Figure 7B). Median values of viable counts of *E. coli* LF82 and *Escherichia* phages (not accounting for samples the where no viable phage was detected) were found in feces at MOI of 0.1, suggesting that *E. coli* typically occurred at higher numbers than phages in the GIT of mice. However, viable *Enterococcus* phages could only be detected at low titers upon phage enrichment with *E. faecalis* OG1RF among a limited number (3/22) of feces samples collected throughout the experiment.

**FIGURE 8:**
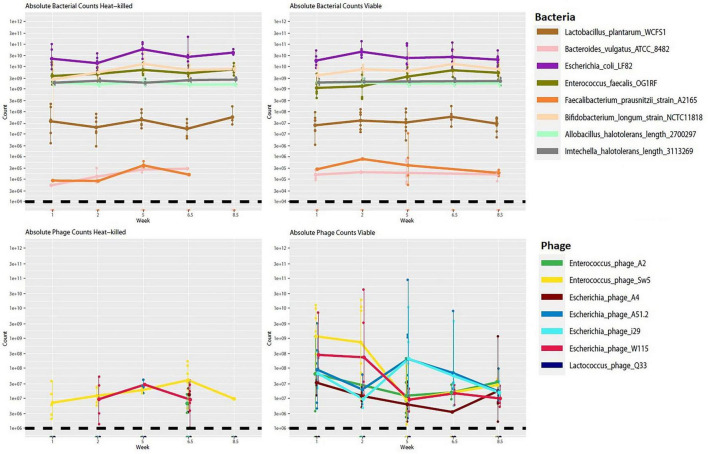
Absolute abundance counts for bacterial and phage genomes across time for viable and heat-killed cohorts. Each point represents absolute genome abundance per sample. Lines are joined at the mean non-zero count for each genome. The black dashed line represents the estimated threshold of detection for hosts and phage, respectively.

**FIGURE 9:**
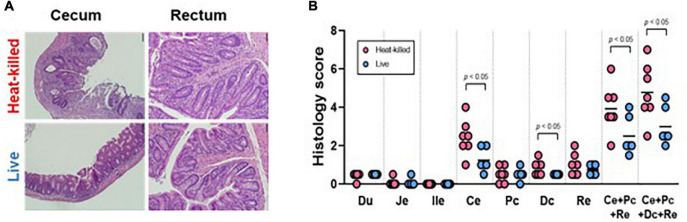
**(A)** Representative histology pictures taken of the cecum and the rectum of mice seeded with the SIHUMI consortium and administered heat-killed or viable EE-phage and **(B)** histology scores of the duodenum (Du), jejunum (Je), ileum end (ile), cecum (Ce), proximal colon (Pc), distal colon (Dc), rectum (Re), combined scores. The bar indicates the mean. A two-tailed unpaired *t*-test was performed, with statistical significance recorded as *p < 0.05.

We also performed short-read shotgun sequencing on DNA extracted from mouse fecal samples over selected time points. Although there was a trend toward a lower abundance of the target hosts (*E. coli* and *E. faecalis*) in the viable cohort relative to the control, no significant changes in bacterial abundance were noticeable across the viable and heat-killed cohorts (Figure 8). We also noted that there were no statistically significant changes in the relative abundance of any of the other community members (Supplementary Figure S10). This result could suggest that the phage cocktail did not interact with the bacterial community, possibly due to reduced activity following passage through the stomach or interaction with the mouse immune system. However, an observed increase in the number of phage genomes detected in the test cohort, along with apparent blooms of individual phage across time (Figure 9), suggests that phage replication and host interaction were occurring in the viable cohort. However, the longitudinal phage-host dynamics observed in the viable cohort indicate that this interaction did not significantly affect bacterial host abundance.

We used germ-free IL-10–/– mice to determine if phage treatment could impact markers of inflammation. No statistically significant differences were observed in cytokine measurements of unstimulated colorectal colonic strip cultures (IFN-?/IL12p40) from both cohorts (Supplementary Figure S11). Furthermore, no statistical difference was observed in the histology scores of the duodenum, jejunum or ileum samples from both cohorts. However, histology scores from the cecum and distal colon samples and the combined scores from the test cohort exhibited a statistically significant reduction in colitis phenotypes compared to controls (Figures 9A,B).

## Discussion

In this study, we isolated lytic phages targeting *E. coli* and *E. faecalis* to create a phage cocktail to assess phage-targeted knockdown of these species within a bacterial consortium. The main objective of this study was to gain insights into the potential of phages as tools for microbiome engineering of these species, as both are implicated as contributors to the onset of IBD (Darfeuille-Michaud et al., 1998; Balish and Warner, 2002; Kotlowski et al., 2007).

Three different phage types at the genus level (*Vequintavirus, Krischvirus &* Rak2-like) were isolated against *E. coli*. These phages were found to collectively infect 35% (12/34) of tested isolates of this species. *E. coli* associated with the onset of IBD belong to the phylotypes of D, B2 and F [32,73,74]. Of the isolates tested, phages formed plaques on 35% (6/17) and 43% (3/7) of B2 and D phylogroup strains, respectively. Limited description is available that details *E. coli* phage susceptibility regarding phylotyping. However, the sensitivity of bacterial isolates to phages of study is not dissimilar from other reported *Escherichia* Rak2-like phages and phages of the genera *Vequintavirus* and *Krischvirus*, infecting between 15 and 30% of total tested strains assessed (Korf et al., 2019). These phages were found to possess limited infectivity against a panel of *E. coli* isolates obtained from individuals with IBD, infecting only 6 of 17 of such isolates. This finding suggests that a broader cocktail of phages might be required to target all isolates of this bacterium among different individuals. These phages were also found to infect *E. coli* phylotypes (A & E) that are not typically associated with IBD. *Enterococcus* phages could collectively infect 80% of the 20 *E. faecalis* isolates tested. Of these, nine isolates were from individuals with IBD, of which seven were sensitive to the phage. *Enterococcus* phages of this study belong to the genera *Kochikohdavirus* and *Schiekvirus*, which are often reported to possess an extremely broad host range (Uchiyama et al., 2008; Khalifa et al., 2015). These genera lie within the family *Herelleviridae*, with phages of other taxa within this family infecting other genera, such as *Staphylococcus* and *Listeria*, and often described as having a broad host range (Klumpp et al., 2008; Arroyo-Moreno et al., 2022). As such, phages of this family are frequently employed in phage therapy or biocontrol applications indicating these *Enterococcus* phages would be ideal candidates for microbiome manipulation perspective.

We utilized continuous fermentation as an *in vitro* model for the GIT, which attempts to mimic the passage of nutrients and waste within this environment. Our continuous fermentation demonstrated that our phage cocktail, composed of four different *Escherichia* phages and two *Enterococcus* phages, could cause targeted knockdown of their hosts *E. coli* LF82 and *E. faecalis* OG1RF. The knockdown of these targeted species occurred in the presence of four other bacterial species (*L. plantarum*, *B. vulgatus, F. prausnitzii*, and *B. longum*). Most studies to date have focused on the direct interaction between phage–host pairs without considering the effect that this interaction may have on other community members likely to be present in the GIT. The human gut microbiome has been estimated to contain no less than 160 species per individual (Qin et al., 2010). Utilizing a six species simplified human microbiome consortium (SIHUMI-6) of gut bacteria our *in vitro* model indicates that phage predation can significantly alter the abundance of non-target species. These effects are likely brought about by phage predation reducing the fitness of target bacteria (*E. coli* and *E. faecalis*), leading to increased relative fitness in other community members (*L. plantarum* and *F. prausnitzii*) with putative protective effects. These effects could be due to competition for nutrients or the production of growth-limiting substances. Furthermore, reduced levels of other community members (*B. longum*) may be due to reduced interaction between them and the phage-targeted species, for example, cross-feeding or syntrophic activities relating to metabolites (Seth and Taga, 2014). The results of these fermentations reinforce the findings of Hsu et al. (2019) where it was shown that phages administered to gnotobiotic mice colonized by a defined consortium of bacteria could be observed to cause a reduction in host species that resulted in negative or positive changes to the abundance of other community members. Clearly, understanding the interaction between bacterial communities of the gut microbiome is essential to understanding its functionality and the subsequent impact on human health. However, limited strategies have been developed to study this aspect of the GIT microbiome (Schmidt et al., 2018). Continuous fermentation with bacterial communities treated with phages as described within this study may merit further exploration as an approach to detect interactions between different species of a bacterial community.

This continuous fermentation model also demonstrated the development of phage resistance for both *E. coli* and *E. faecalis* in the presence of other bacteria species. This finding shows that the additional pressure exerted by competing bacteria species of the SIHUMI-6 consortium did not impede the development of phage resistance. The emergence of phage resistance likely explains the noticeable re-growth of *E. coli* in the latter stages of fermentation in the vessel treated with viable phages. Tanji et al. (2005) also observed that, within a continuous fermentation using a monoculture of *E. coli* treated with a three component phage cocktail, re-growth under anaerobic conditions was observed post phage addition.

We also noted that the virulent *Escherichia* phage A4 likely possesses an atypical infection strategy with a lack of observed lysis of the host. We have also worked with another example of phage from this lineage, *Pectobacterium* phage CBB. We also failed to observe lysis due to this phage even though high titers could be achieved (Buttimer et al., 2017). Other studies have also highlighted that some phages can have infection strategies that do not conform to the classic interpretation of the lytic cycle. For example, *Bacteroides xylanisolvens* phage crAss002 is a virulent phage that does not form plaques or cause lysis in broth even at high titers (Guerin et al., 2021). In conclusion, when selecting phages for the gut microbiome engineering, it is simply not enough to choose virulent phages. Attention must also be given to phage infection strategies and their effect on host numbers.

The findings made in our continuous fermentation did not translate to our experiments with gnotobiotic mice colonized with the same SIHUMI-6 consortium and treated with either heat-inactivated or viable phages twice a week over nine weeks. Neither *E. coli* LF82 nor *E. faecalis* showed a statistically significant difference in numbers between treatment and control groups at selected time points across the trial (weeks 0, 2.5, 4.5, and 9). Metagenomic analysis (weeks 1, 2, 6.5, and 8.5) indicated lower levels of these species, but this was not statistically significant, and there were no significant shifts in the levels of the other four community members of the SIHUMI-6 consortium. Also, we could see blooms in phage numbers among viable phage treated mice. However, only viable *Escherichia* phages could be enumerated in mouse feces. They were typically present at an MOI of 0.1 (not accounting for the 54% of samples [*n* = 56] where no viable phage was detected), based on median values of host and phage counted. We also detected low levels (11–33% of inspected colonies) of *E. coli* LF82 resistant to *Escherichia* phages used in this murine study. However, viable *Enterococcus* phages could only be found by phage enrichment with their bacterial host strain among inspected samples.

Three potential outcomes could be anticipated in studies examining the targeted knockdown of bacteria by phages in the gut within murine models. These outcomes are as follows: (i) persistent reduction of host numbers throughout the trial, (ii) a transient reduction or (iii) no impact observed on host numbers. In previous trials, these effects were observed using *E. coli* (Tanji et al., 2005; Maura et al., 2012b) and *E. faecalis* (Duerkop et al., 2016). Additionally, where no impact on host numbers is identified, phage numbers can rapidly decrease post a single dose. However, an alternative situation can arise where phage and host co-exist, whereby the phage replicates on the host in a manner that does not result in its eradication from the murine gut (Maura et al., 2012b). These observations likely correlated with how virulent phage infects the host (i.e., capable of host lysis in broth) and the number of phages present relative to the host in the gut.

The pharmacokinetics of phage infection in the gut has been considered by Danis-Wlodarczyk et al. (2020). There is likely a correlation between the observed knockdown of host levels and the initial phage load that reaches the gut. In one scenario, the number of phages that reaches the gut is low (below the inundation threshold), but phage presence in the gut is maintained. In this situation, there will only be numbers of phages sufficient to encounter its bacterial host to replicate and maintain their levels, enabling persistence and mitigating the dilution effect of phage numbers in this location due to the passage of feces in the gut. The anatomy of the digestive tract and the spatial distribution of microbes also plays a role in the probability of a phage coming into contact with its host (Lourenço et al., 2020). Phages infecting bacterial species of the GIT have likely evolved to replicate at a rate that prevents host eradication in this environment. If phage replication exceeds that of the host, this would eventually lead to the eradication of the bacterial host and subsequently the phage. However, in another scenario, initial phage contact could occur at a level where phage numbers exceed those of the target bacterium (above inundation threshold) before the dilution effect of GIT has sufficient impact on reducing their level. In that case, one could expect a reduced load of the host. For example, Maura et al. (2012a) found an improved reduction of host levels in ilea of mice when given a 100 fold higher titer of phage, implying that the load of phage that initially reaches the gut is essential to see the targeted knockdown of bacteria. Additionally, murine trials that use a single dose of phage have reported a transient target reduction, and suppression can be maintained when conducted with repeated doses of phage (Tanji et al., 2005), which was performed in this study. Furthermore, numerous murine trials that report a reduction of host bacteria upon treatment of phage treat mice apply an antacid to reduce stomach pH to ensure sufficient phage numbers reach the GIT (Reyes et al., 2013; Hsu et al., 2019). This practice likely ensures that phage numbers reaching the GIT exceed the inundation threshold. We did not perform this measure for the murine trial described in this study.

Cytokine measurements (IFN-?/IL12p40) conducted on unstimulated colorectal colonic strip cultures showed no differences between cohorts of mice treated with either viable or heat killed phages. This finding contrasts with histology score differences between the cecum and distal colon samples and the combined GIT scores between these cohorts, showing significantly reduced colitis phenotypes. It is difficult to explain the latter finding without differences being detected in the composition of bacterial consortia between cohorts. This effect may have resulted from a functional difference in the SIHUMI-6 consortia between treatment groups. However, examination of viral and bacterial contigs derived from metagenomic sequencing for single nucleotide polymorphisms (SNPs) and structural variants did not give any useful insight into these histology scores (data not shown).

In conclusion, one potential reason for the discrepancy between the performance of the phage cocktail used for targeted knockdown of *E. coli* and *E. faecalis* within a bacterial consortium in continuous fermentation vs. *in vivo* within this study may relate to the number of phages that successfully manage to reach the GIT of mice. Our understanding of how phages interact within *in vivo* gut models remains rudimentary and so it is essential to report findings where no host targeted knockdown was observed, which may otherwise be regarded as simply failed experiments.

## Data availability statement

The datasets presented in this study can be found in online repositories. The names of the repository/repositories and accession number(s) can be found in the article/Supplementary Material.

## Ethics statement

The studies involving human participants were reviewed and approved by Cork Research Ethics Committee. The patients/participants provided their written informed consent to participate in this study. The animal study was reviewed and approved by Institutional Animal Care and Use Committee of the University of North Carolina at Chapel Hill.

## Author contributions

CB, AS, and JC: methodology. JC, CB, AB, TS, EM, PB, LS, AO, BL, and JH: formal analysis. JC, CB, AB, TS, EM, PB, LS, AO, BL, and JH: investigation. RS and CH: resources. CB and TS: writing—original draft preparation. CB, TS, LD, CH, and RS: writing—review and editing. CH and RR: supervision. CH and LD: project administration. All authors contributed to the article and approved the submitted version.

## Conflict of interest

The authors declare that the research was conducted in the absence of any commercial or financial relationships that could be construed as a potential conflict of interest.

## Publisher’s note

All claims expressed in this article are solely those of the authors and do not necessarily represent those of their affiliated organizations, or those of the publisher, the editors and the reviewers. Any product that may be evaluated in this article, or claim that may be made by its manufacturer, is not guaranteed or endorsed by the publisher.
