# Supplementary material for: Impact of a phage cocktail targeting Escherichia coli and Enterococcus faecalis as members of a gut bacterial consortium in vitro and in vivo
Source: Front Microbiol. 2022 Jul 22;13:936083. doi: 10.3389/fmicb.2022.936083 (PMC9355613; doi:10.3389/fmicb.2022.936083)
Supplement: Supplementary file 1 [file Data_Sheet_1.docx]

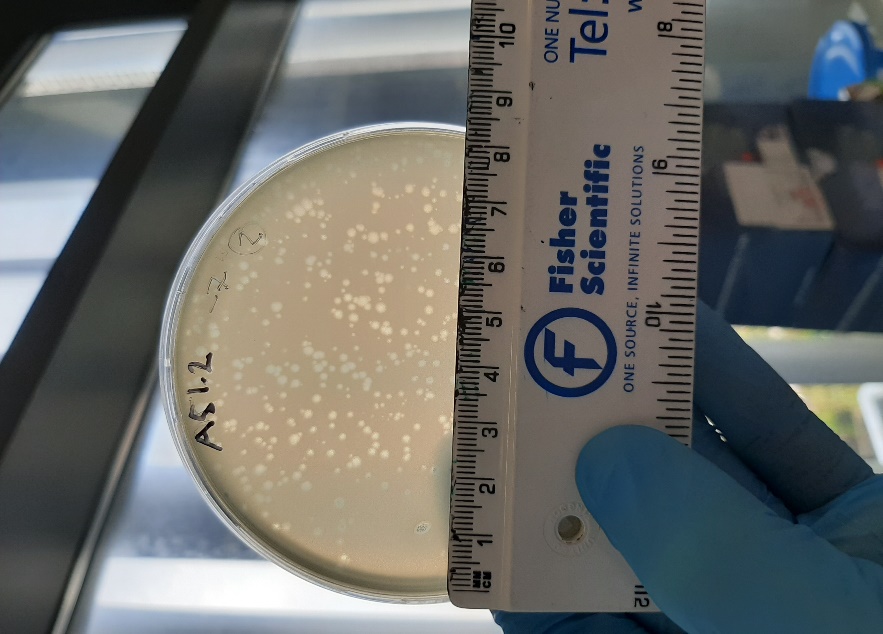

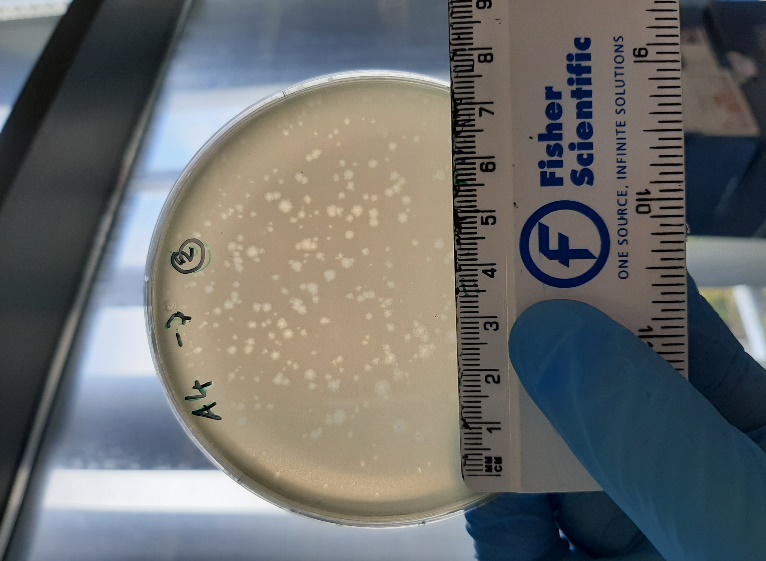

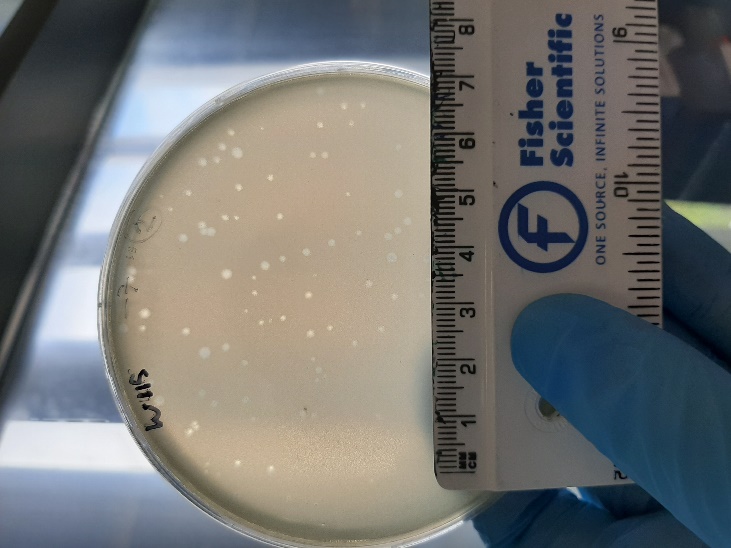

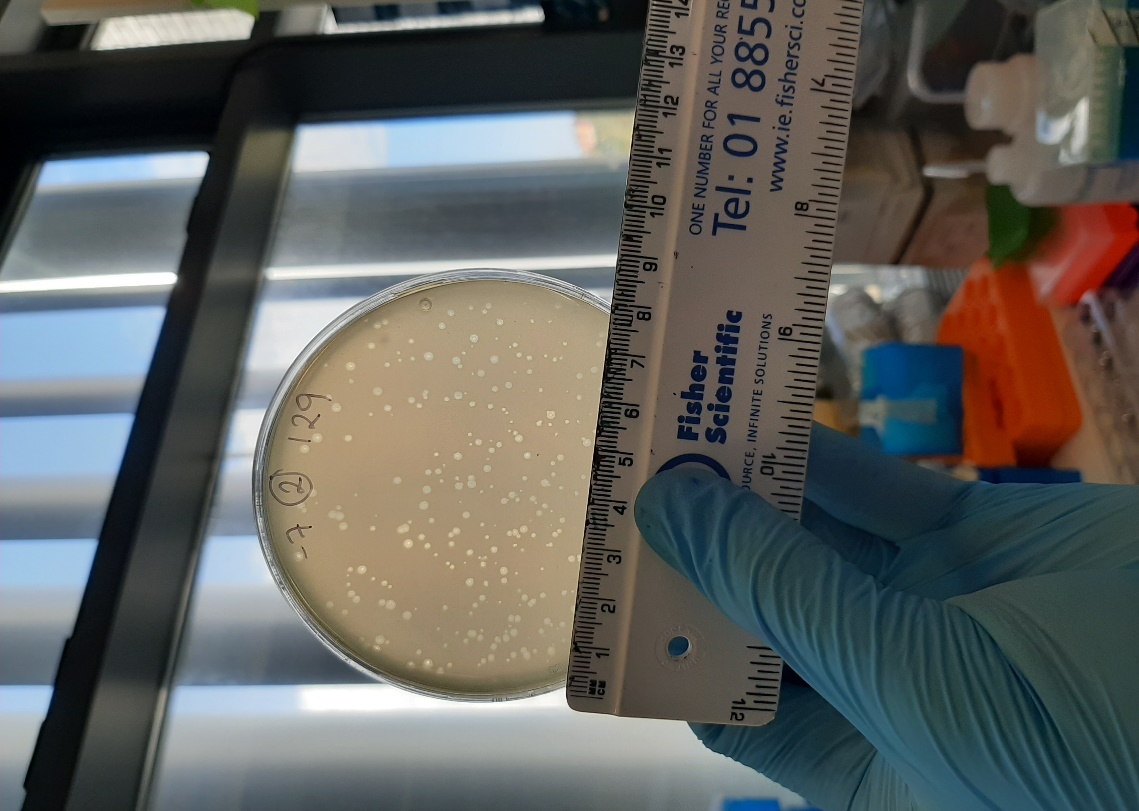

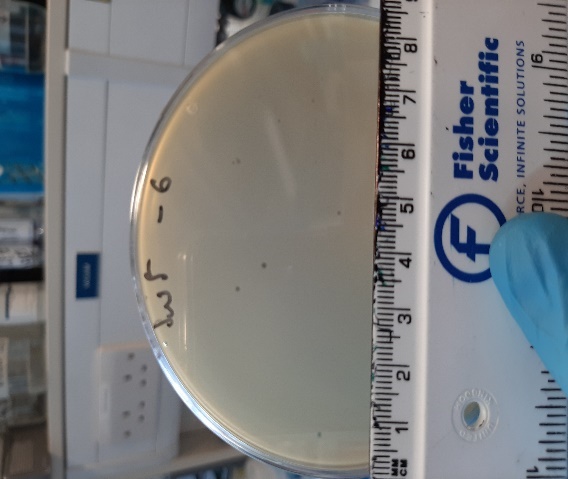


Figure S1. Plaque morphology of *Escherichia* phages (on *E. coli* LF82) and *Enterococcus* phages (on *E. faecalis* OG1RF) using an overlay of 0.2% (w/v) agarose.


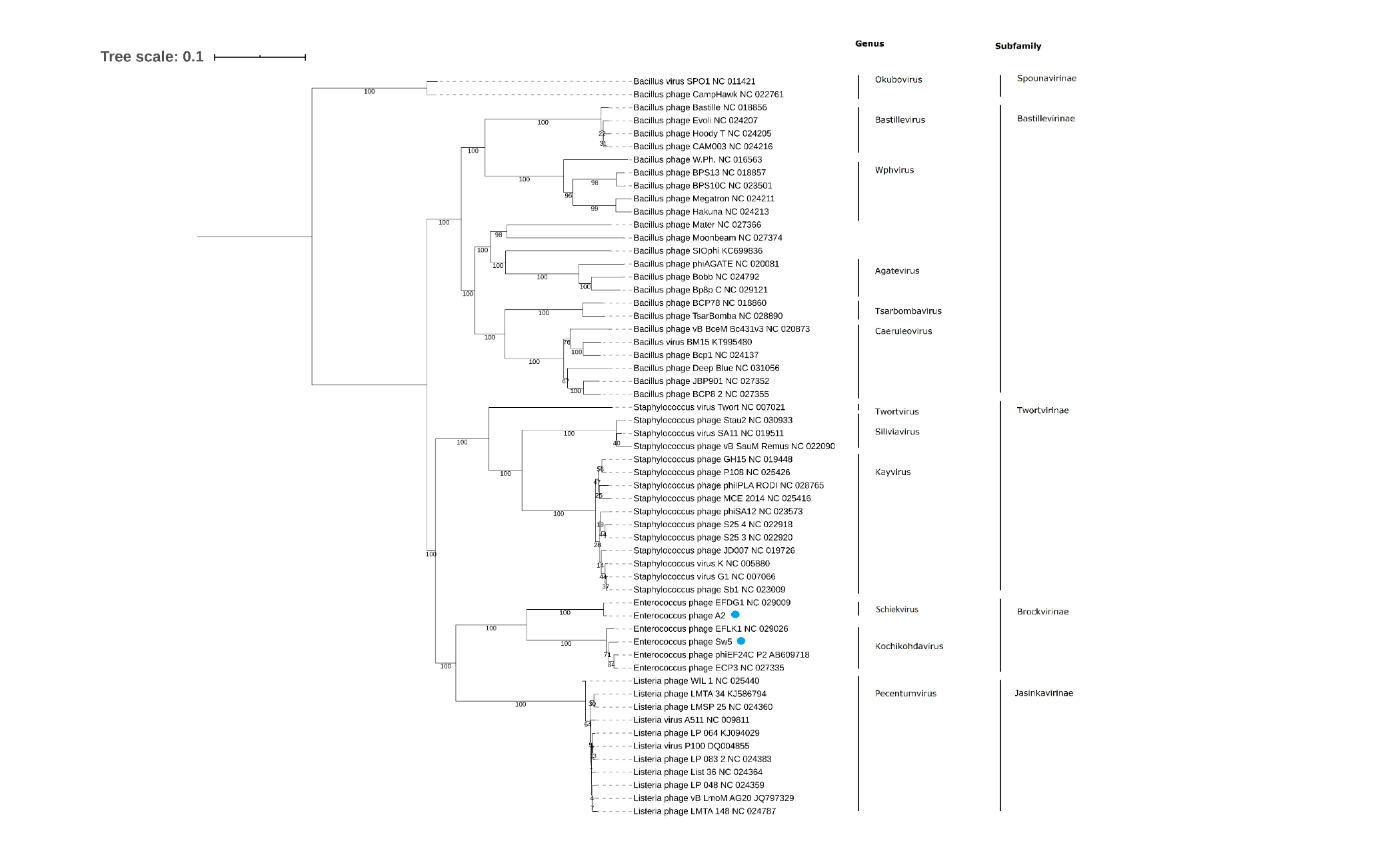


Figure S2. Phylogenetic tree of EE phages. Amino acid-based VICTOR-generated phylogenomic Genome-BLAST Distance Phylogeny (GBDP) tree of *Enterococcus* phages Sw5 and A2 (highlighted with a blue dot), with members of the *Herelleviridea* family Inferred using the formula D4 and yielding average support of 68%. The numbers below branches are GBDP pseudo-bootstrap support values from 100 replications. Established genera and subfamilies are highlighted


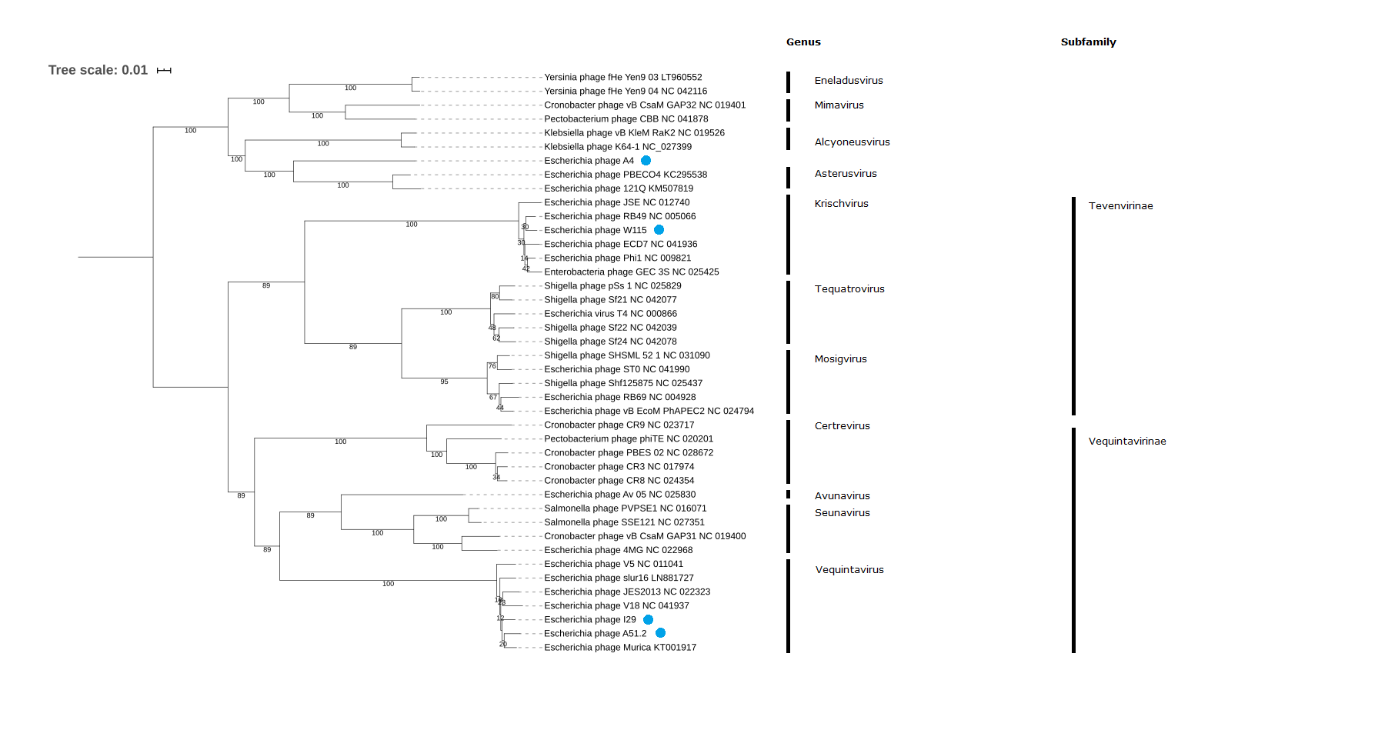


Figure S3. Phylogenetic tree of EE phages. Amino acid based VICTOR-generated phylogenomic Genome-BLAST Distance Phylogeny (GBDP) tree of *Escherichia* phages W115, I29, A51.2 and W115 (highlighted with a blue dot), with members of the *Myoviridae* family Inferred using the formula D4 and yielding average support of 72%. The numbers below branches are GBDP pseudo-bootstrap support values from 100 replications. Established genera and subfamilies are highlighted


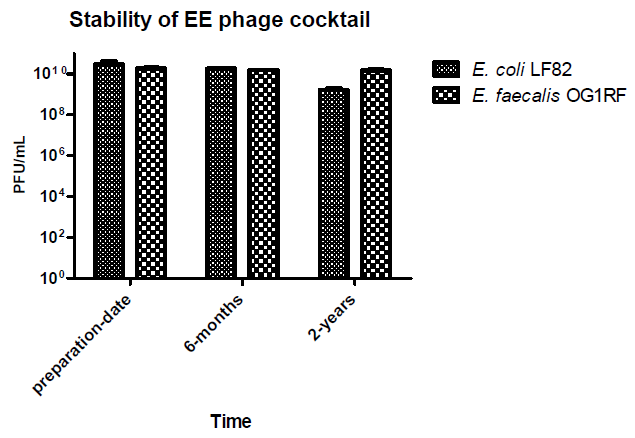


Figure S4. Inspection of stability of EE phage cocktail (in SM buffer), when stored at 4 °C over a period of six months & 2 years by spot assay on host strains *Escherichia coli* LF82 and *Entercoccus faecalis* OG1RF.


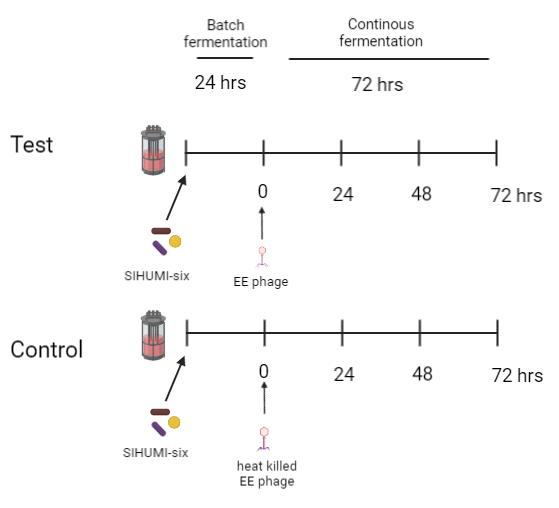


Figure S5. Diagram illustrating the experimental setup of the fermentations conducted in this study. Fermentation vessel inoculated with SIHUMI-6 consortium and grown as a batch fermentation for 24 hrs. After which either viable or heat killed EE phage cocktail was added and the fermentation was switched to a continuous fermentation format. During this time frame samples were collected at 0, 24, 48 and 72 hrs.


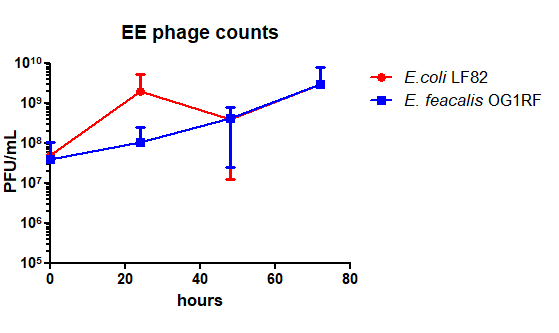


Figure S6. Enumeration by of EE phages by spot assay during continuous fermentation at different time points treated with heat-killed or viable phage (bars represent mean ± SD).


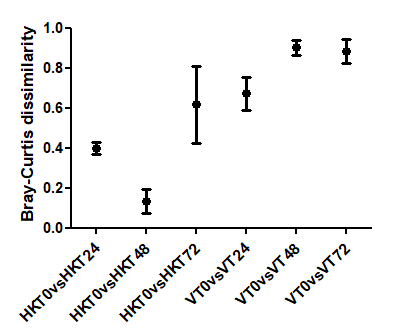


Figure S7. Dissimilarity between time points 24 hrs, 48 hrs & 72 hrs vs 0 hrs among continuous fermentations with SIHUMI-6 consortia treated with either heat killed (HK) or viable (V) EE phages.


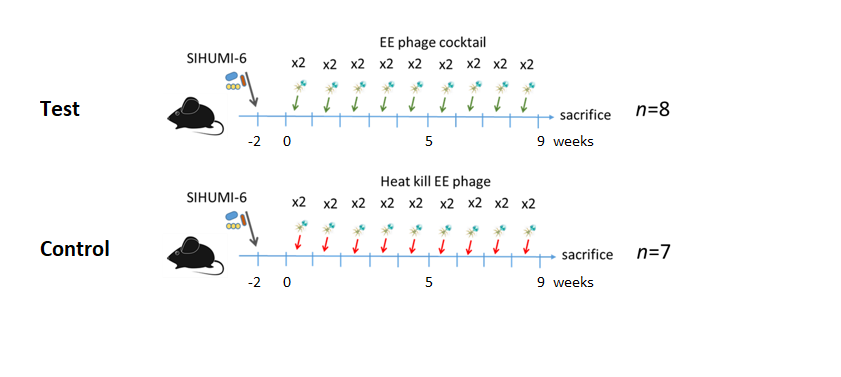


Figure S8. Diagram illustrating the experimental setup of the murine trail of this study, indicating time points at which mice were seeded with the SIHUMI-6 consortium and administered the EE phage cocktail.


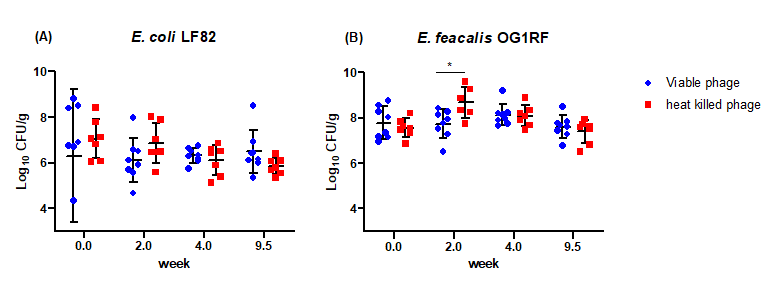


Figure S9. (A) Counts of *E. coli* and (B) *E. faecalis* in faeces of mice colonised with the SIHUMI-6 consortium and treated with either the viable or heat treated EE phage cocktail. Bars represent mean ± SD.


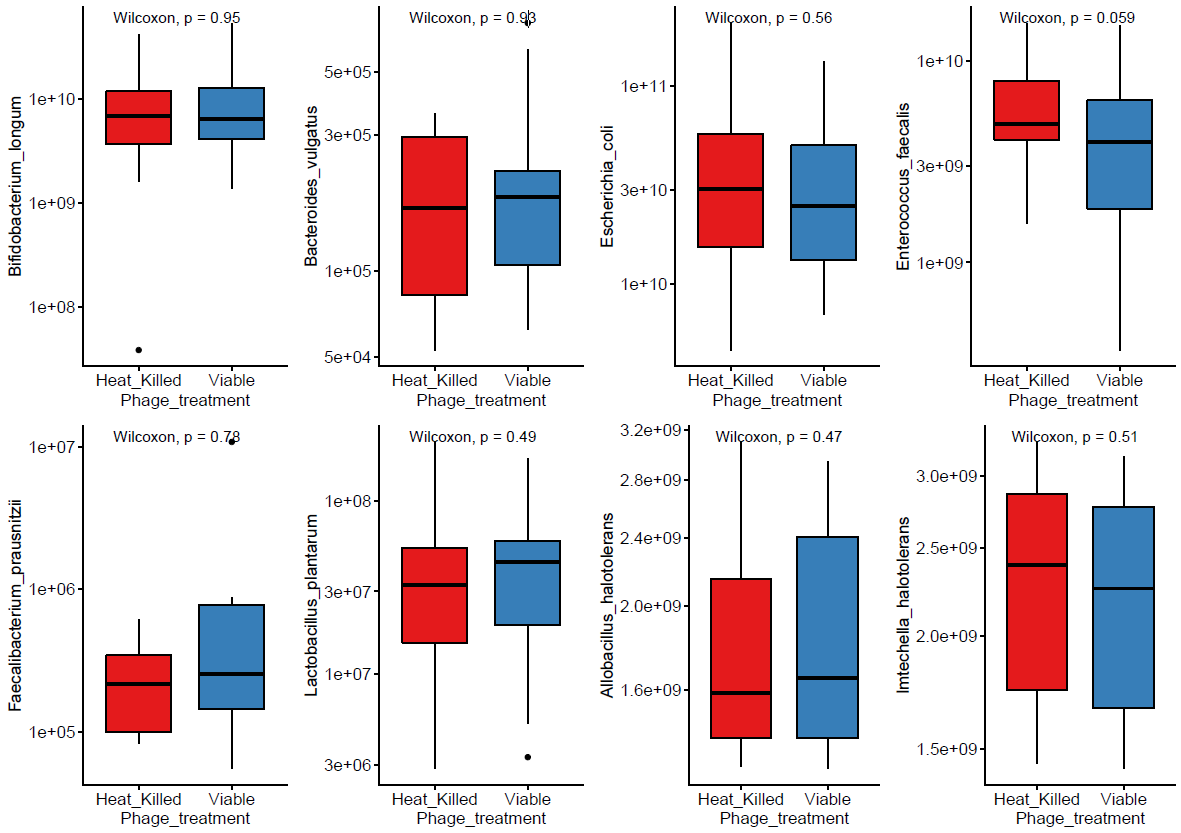


Figure S10. Absolute counts of bacterial genomes across viable and heat-killed cohorts. Wilcoxon test employed for tests of significance.


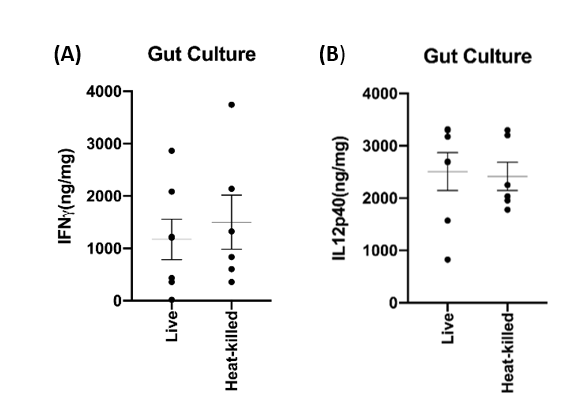


Figure S11. Cytokine measurements of unstimulated colorectal colonic strip cultures of cytokine measurements of (A) IFN-ƴ and (B) IL12p40 taken from germ free mice cohorts seeded with a bacterium consortium (*E. coli*, *E. faecalis*, *L. plantarum*, *B. vulgatus*, *F. prausnitzii* & *B. longum*) and where mice where either treated viable or heat activated phage cocktail targeting *E. coli* and *E. faecalis*.
